# EUS-Guided Diagnosis of Gastric Subepithelial Lesions, What Is New?

**DOI:** 10.3390/diagnostics13132176

**Published:** 2023-06-26

**Authors:** Thomas Vasilakis, Dimitrios Ziogas, Georgios Tziatzios, Paraskevas Gkolfakis, Eleni Koukoulioti, Christina Kapizioni, Konstantinos Triantafyllou, Antonio Facciorusso, Ioannis S. Papanikolaou

**Affiliations:** 1 Hepatology and Gastroenterology Clinic, Charité Campus Mitte, Charitéplatz 1, 10117 Berlin, Germany; 2 1st Department of Internal Medicine, 251 Hellenic Air Force & VA General Hospital, 3 Kanellopoulou Str., 11525 Athens, Greece; 3 Department of Gastroenterology, “Konstantopoulio-Patision” General Hospital, 3–5, Theodorou Konstantopoulou Str., Nea Ionia, 14233 Athens, Greece; 4 Hepatogastroenterology Unit, Second Department of Internal Medicine-Propaedeutic, Medical School, National and Kapodistrian University of Athens, 12462 Athens, Greece; 5 Department of Medical Sciences, University of Foggia, Section of Gastroenterology, 71122 Foggia, Italy

**Keywords:** keyword, gastric subepithelial lesions, endoscopic ultrasound, EUS elastography, contrast-enhanced EUS, EUS-AI

## Abstract

Gastric subepithelial lesions (SELs) are intramural lesions that arise underneath the gastric mucosa. SELs can be benign, but can also be malignant or have malignant potential. Therefore, correct diagnosis is crucial. Endosonography has been established as the diagnostic gold standard. Although the identification of some of these lesions can be carried out immediately, solely based on their echo characteristics, for certain lesions histological examination is necessary. Sometimes histology can be inconclusive, especially for smaller lesions. Therefore, new methods have been developed in recent years to assist decision making, such as contrast enhanced endosonography, EUS elastography, and artificial intelligence systems. In this narrative review we provide a complete overview of the gastric SELs and summarize the new data of the last ten years concerning the diagnostic advances of endosonography on this topic.

## 1. Introduction

Gastric subepithelial lesions (SELs) are intramural lesions that arise from the layers underneath the gastric mucosa (muscularis mucosae, submucosa, muscularis propria, and rarely from the serosa). They are discovered mainly during upper GI endoscopy performed for other indications with an incidence of 0.36% of upper GI endoscopies, as showed in 1991 [1]. To our knowledge, no study assessing their incidence has been published since then. We estimate at least a slightly higher incidence, as the better image quality of the new endoscopes would lead to higher detection rates. 

SELs are mostly incidental findings, as most of them are asymptomatic. Bigger lesions, however, can cause dysphagia, overt or occult gastrointestinal (GI) bleeding, and chronic anemia [2]. SELs may be nonneoplastic, neoplastic but benign, neoplastic with malignant potential, or malignant [2]. Only 15% of the SELs are malignant at the time of diagnosis [3]. Therefore, their correct identification is of vital importance for a successful management. Additionally, these lesions need to be differentiated from masses that cause extrinsic compression of the gastric wall and from epithelial lesions that mimic SEL. 

Due to their location, standard luminal endoscopy cannot determine the exact nature of these lesions. Therefore, endosonography (EUS) alone or EUS with fine needle aspiration (FNA) or fine needle biopsy (FNB) have been established as the next step in the diagnostic algorithm [2,4]. Although these methods have a high level of accuracy, sensitivity, and specificity for lesions > 2 cm [5,6], the correct diagnosis of SELs, especially those of ≤2 cm, still poses a challenge. 

With this review we aim to provide a practical overview of all SELs, including both the most common and rare SELs, and to illustrate the new data of the last ten years concerning the diagnostic advances of endosonography and diagnostic algorithms in this topic, in order to assist gastroenterologists and endoscopists in their clinical decision making. 

## 2. Methods

For this narrative review we searched articles in the PubMed Database published in the last decade until January 2023, in order to assess the latest advancements in this area. Firstly, we performed a broad search on the topic using the following keywords: “gastric” or “stomach”, “subepithelial lesions” or “submucosal lesions”, “gastrointestinal stromal tumors”, “endosonography”. Subsequently, we performed a focused search on specific diagnostic advances of EUS on this topic by adding the keywords: “fine needle aspiration”, “fine needle biopsy”, “contrast enhanced endosonography”, “EUS Elastography”, and “Artificial Intelligence in EUS”.

## 3. Types of Lesions

### 3.1. Extrinsic Compressions

When evaluating a gastric subepithelial lesion, endoscopists need to firstly differentiate if the lesion is intra- or extramural. Common structures that can cause an extrinsic compression are the xyphoid bone (fundus), the left hepatic lobe, spleen/accessory spleen (fundus, upper body), the gallbladder (antrum), or pathologic abdominal masses (such as tumors, pancreatic pseudocysts, enlarged lymph nodes) and vessel aneurysms [2,7,8]. Endoscopists can achieve a 92% sensitivity of recognizing extrinsic compressions using EUS [9]. For better differentiation between intra- and extramural lesions, a combined approach of using both frequencies of 7.5 MHz and higher frequencies of 12 MHz has been proposed. Using 7.5 MHz, a deeper view can be obtained and the correlation between the gastric wall and the lesion can be assessed better, whereas using 12 MHz provides a more detailed image of the interface between the gastric serosal wall and the extramural lesion [10].

### 3.2. Subepithelial Lesions

There are many types of gastric SELs. The most common are summarized in Table 1. Mesenchymal tumors are the most frequent ones, comprising 54% of the gastric SELs [3,11]. The majority of them are gastrointestinal stromal tumors (GISTs) and leiomyomas [12]. Other common SELs are schwannomas (less common mesenchymal tumors arising from the nerve sheath), lipomas, pancreatic rests (heterotopic pancreas tissue), carcinoid tumors (neuroendocrine tumors), varices, duplication cysts, and inflammatory fibroid lesions [3]. Less common lesions include lymphoma, lymphangioma, glomus tumor, and submucosal metastases [3,10]. Exact epidemiological data for each lesion are lacking due to their rarity. 

Apart from the abovementioned lesions, we need to point out that there are more gastric SELs, which are rare and thus are not portrayed in the guidelines [2,4]. These are mesenchymal lesions such as sarcomas, hemangiomas, inflammatory myofibroblastic tumors, plexiform myxofibromas, calcifying fibrous tumor, and desmoid fibromatosis [13]. A rare entity is also a gastric wall abscess [14]. Despite their low incidence we believe that clinicians should be aware of these conditions. Therefore, we summarize the number of known cases and their echo characteristics, as well as information about their malignant potential, the need for diagnostic biopsy, and the respective immunohistochemical test in Table 2. 

**Figure 1:**
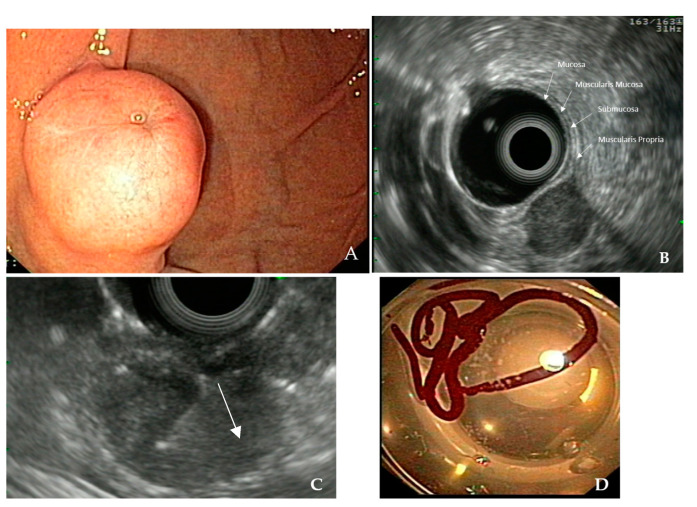
Subepithelial lesion at the gastric body. (**A**) Endoscopic image, note the small erosion on the tip of the lesion. (**B**) Radial EUS of the lesion; size approximately 15 × 15 mm, note that the lesion arises from the muscularis propria, is hypoechoic, and slightly heterogenous with sharp border. DD GIST, less likely schwannoma or leiomyoma. (**C**) EUS-FNB of the lesion with radial EUS. Note the needle (arrow) inside the lesion. (**D**) Specimen obtained; histological assessment verified the suspected diagnosis of a gastric GIST.

For the differential diagnosis of all the above lesions, endoscopists need to describe the following echo characteristics: 1. Wall layer of origin. This is best determined by focusing on the transition zone between the normal gastric wall and the SEL. 2. Echogenicity and homogeneity of the lesion. 3. The presence of vessels by using doppler imaging. 4. The regularity of the borders. 5. Size. 6. Signs of infiltration of adjacent organs and lymph nodes [10]. Based on this information, endoscopists can already identify the following lesions: duplication cysts, varices, ectopic pancreas, and lipomas [2]. For the rest of the SELs, histology is required. Figure 1 demonstrates an example of a subepithelial gastric lesion. 

A special category of subepithelial lesions in terms of diagnosis are neuroendocrine tumors (NETs). Since NETs can arise from the muscularis mucosa, most times, a forceps biopsy can lead to diagnosis. When the lesion lies completely in the submucosal layer, then an EUS-FNB is necessary [15]. No data were found in the literature that determine the frequency of NETs lying only in the submucosa. Additionally, to the authors’ knowledge, there has not yet been a study published that assesses the accuracy of EUS-guided diagnostics specifically for NETs. Primarily, the role of EUS in the diagnosis of gastric NETs is the assessment of the depth of invasion and the presence of enlarged lymph nodes, so that the treatment strategy can be determined [16]. 

### 3.3. Epithelial Lesions Mimicking SELs

Last but not least, we want to emphasize that early gastric cancer may rarely mimic subepithelial lesions having intact epithelium. An older study found five gastric cancers as subepithelial masses, and at least three recent case reports have published similar cases [11,17,18]. The right diagnosis was achieved either at time of presentation or during follow-up. This shows the importance of reaching the right diagnosis at initial presentation, and, in case of inconclusive findings, the significance of scheduling follow up examinations.

**Table 2:** Overview of rare gastric SELs. EGD: esophagogastroduodenoscopy, IHC: Immunohistochemistry, NR: not relevant [13,14,19,20,21,22,23,24,25,26,27,28,29,30].

Type	Origin	EUS Morphology	Malignant Potential	Biopsy Needed	IHC	No. Cases
Sarcoma (leiomyosarcoma, liposarcoma, synovial)	2nd, 3rd, 4th	Nonspecific	NR	Yes	CD117− Desmin+ SMA+	100, 38, 36 respectively
Hemangioma	3rd, 4th	EGD: bluish black intramural lesion EUS: similar to GIST, calcifications, Doppler signal in case of cavernous transformation	Rarely	No	NR	At least 70
Inflammatory myofibroblastic tumor	4th	Hypoechoic, heterogenous, ill-defined borders, oval shape	Rarely	Yes	SMA+, ALK, CD34+	19
Plexiform myxofibroma	3rd	Hypoechoic, homogenous, irregular shape, well-defined borders, possible mucosal invasion with ulceration, almost always in the antrum	No	Yes	SMA+, CD117− Vimentin+	Approx. 130
Desmoid fibromatosis	4th	Hypoechoic, well-defined borders	No	Yes	b-catenin+ SMA+, CD117−	9
Calcifying fibrous tumor	All, mostly 3rd	Heterogenous, mostly iso-hypoechoic calcifications, well-defined borders	No	Yes	Various markers 25% Vimentin+	Approx. 300
Gastric wall abscess	3rd, 4th	Hypoechoic, heterogenous	No	No	NR	Approx. 500
Idiopathic granulomatous gastritis	4th	Hypoechoic mass	No	Yes	Multinodular Granulomas	<50
Lipomatous hemangiopericytoma	Not clearly stated	Hyperechoic, oval shape, well-defined borders	Unclear	Yes	CD34+ CD99+ Vimentin+	1

## 4. Tissue Acquisition 

### 4.1. Indications

In case the lesion cannot be diagnosed based on solely its echo characteristics, then tissue acquisition is necessary. For example, GISTs, leiomyomas, schwannomas, granular cell tumor, and lymphoma can originate from the muscularis propria (4th layer) and are hypoechoic on EUS. Given the malignant potential of some of these lesions, and especially GISTs, histological examination is considered mandatory [31]. According to the guidelines, EUS-guided sampling is indicated for hypoechoic SELs > 2 cm, as below this limit, the risk of malignancy is very low. For small lesions (≤2 cm), biopsy, resection, or surveillance is recommended; this decision should be made on an individual basis [2,4]. Furthermore, histological examination is also indicated in cases of lesions with suspicious features for malignancy on EUS or endoscopy, regardless of their size. These features include presence of ulceration in luminal endoscopy, irregular border, echogenic foci, cystic spaces, and adjacent lymph node enlargement [32,33,34].

Needless to say, EUS-guided tissue acquisition should be performed only when the histologic findings will affect the choice of treatment. This can be achieved ideally by fine-needle biopsy (FNB) or by fine-needle aspiration (FNA), if FNB is not available, as well as other biopsy methods. 

### 4.2. EUS-Guided Fine-Needle Biopsy

In order to overcome the high variability of the EUS-FNA diagnostic yield, fine-needle biopsy (FNB) needles have been introduced. FNB needle types include Franseen-type needle, reverse bevel needle, and fork-tip needle, and can obtain larger tissue samples preserving tissue architecture. EUS-FNB has a very high diagnostic accuracy in detecting GISTs (89–93.8%) vs. EUS-FNA (37–75%) [35,36]. Furthermore, the literature shows that EUS-FNB compared to EUS-FNA leads to better tissue acquisition and higher diagnostic accuracy for all gastric SELs, while lower number of needle passes are needed (mean number of passes 1.73 vs. 2.51) [6,37,38]. It should be noted that in some cases FNB was superior even when FNA was combined with a rapid onsite evaluation of the adequacy of the acquired tissue specimen by a pathologist (ROSE) [37]. 

The higher efficiency of EUS-FNB compared to EUS-FNA in the diagnosis of gastric SELs is observed with all the available needle types. However, direct comparisons between the FNB needle types for the diagnosis of gastric SELs are scarce, as most studies compare the diagnostic performance of FNB needles on mixed groups of lesions (both solid gastrointestinal and pancreatic lesions) or are noncomparative studies. Regarding gastric SELs, EUS-FNB with reverse bevel needle offers a diagnostic rate of 75–91% [39,40], Franseen-type needle 85–94%, even in cases of SELS < 20 mm [41,42], and EUS-FNB with fork tip had an overall accuracy between 84% and 89% in the diagnosis of suspected GISTs with a notable 89% and 84% accuracy in lesions with size 16–20 mm and 11–15 mm, respectively [43]. In another study, the diagnostic yield of EUS-FNB with fork tip for lesions measuring < 20 mm even reached 100% [36]. This is of vital importance, because even small tumors might have malignant features requiring precise diagnosis [44].

To our knowledge, there is only one meta-analysis that compared two of the available FNB needles. It demonstrated that the Franseen needle had a significantly higher percentage of obtaining an adequate sample compared to fork-tip needle (97.6% versus 90.5%, *p* = 0.006) [38]. However, most of the data in this meta-analysis arise from single-cohort and noncomparative studies. An additional study found no difference in diagnostic accuracy between Franseen and reverse bevel needle [42]. The available literature shows that there are not enough data to prove the superiority of one needle type vs. another. Therefore, current guidelines do not recommend a specific type of FNB needle. 

The existing guidelines also reflect the abovementioned data regarding the diagnostic value of EUS-FNB in the diagnosis of gastric SELs. The American College of Gastroenterology (ACG) recommends as a first diagnostic step EUS-FNB or EUS-FNA with ROSE, where EUS-FNB is not available [2]. The European Society of Gastrointestinal Endoscopy (ESGE) considers EUS-FNB as first choice along with mucosal incision-assisted biopsy (MIAB) for the diagnosis of SELs [4]. On the contrary, the American Gastroenterological Association (AGA) suggests EUS-FNB and EUS-FNA equally to determine if a lesion is GIST or leiomyoma [32]. The American Society for Gastrointestinal Endoscopy (ASGE) suggests that EUS-FNB can be used when a definite diagnosis with EUS-FNA is not possible [45], but this recommendation cannot be considered current, as it was published six years ago.

Since the ROSE process is not widely available due to institutional limitations, another approach called macroscopic on-site evaluation (MOSE) has also been proposed. In this approach, the adequacy of the obtained tissue sample is assessed by the endosonographer. According to Iwashita et al., who firstly described the MOSE technique, a macroscopic visible core (MVC) ≥ 4 mm makes the specimen acceptable [46]. Several studies have shown that FNB combined with MOSE required fewer needle passes compared to FNB alone, but had similar diagnostic accuracy, making MOSE a reliable alternative to ROSE [47,48,49]. This is particularly important in cases where the presence of a cytopathologist is not feasible.

### 4.3. EUS-Guided Fine Needle Aspiration 

Although EUS-FNB is the standard of care now, EUS-guided FNA is still an option in the current guidelines if EUS-FNB is not available. The diagnostic accuracy of EUS-FNA for recognizing gastric SELs varies from 60 to 90% [5,50,51]. This variability results from many factors such as tumor size, experience of the endosonographer, location of the target lesion, and presence of an onsite cytopathologist, but not from the size of the needle used. 

Akahoshi et al. found that EUS-FNA was diagnostic in 71% of tumors < 2 cm, 86% of tumors 2–4 cm, and 100% of tumors > 4 cm, concluding that an adequate sample for analysis is more likely to be obtained as the size of the mass increases [52]. Regarding location, SELs located in the antrum are associated with lower diagnostic rates, because the antral wall is thicker and is not easily accessible [53,54]. As expected, large needles (19-gauge vs. 20-gauze, 22-gauge, and 25-gauge) have the potential to obtain larger tissue samples, maintaining tissue architecture. Nevertheless, studies showed no effect of the needle size on diagnostic accuracy of EUS-FNA [51,55,56]. On the contrary, EUS-FNA combined with ROSE can improve the diagnostic yield by 20% [57,58]. In a study of 139 mesenchymal tumors sampled via EUS-FNA, accurate classification of the tumor was more likely to occur in cases when ROSE was performed [36]. Therefore, the American guidelines recommend that FNA is combined with ROSE, if available [2].

### 4.4. Other Biopsy Methods

Additional to FNB and FNA, other biopsy methods such as mucosal-incision-assisted biopsy (MIAB) and single-incision needle-knife biopsy (SINK) have been developed in order to optimize the diagnosis of SELs. These methods can be an alternative to FNA/FNB if they fail to reach a definite diagnosis [2]. The salient point of these “open biopsy” methods is that the lesion is clearly visible after incision of the covering mucosa. Then, direct samples are acquired by biopsy forceps [43,59]. A recent randomized controlled trial with 47 patients and a retrospective study with 177 patients showed that MIAB had a greater diagnostic yield than EUS-FNA and EUS-FNB for SELs < 20 mm, but at the cost of longer procedural time [59,60]. Thus, MIAB can be a reliable choice for tissue sampling, especially in SELs measuring < 20 mm [4]. 

### 4.5. Forward-Viewing vs. Oblique-Viewing Echoendoscope

Regarding the dilemma of which echoendoscope to use (forward-viewing vs. oblique-viewing), there is no clear superiority of one type over the other [2]. Although the diagnostic yield is similar regardless of which echoendoscope is being used, by approaching the gastrointestinal wall vertically with the forward-viewing type, the puncture of the targeted lesion is easier to perform. This leads to the acquisition of larger tissue samples, even in cases of small SELs, and also leads to reduced examination time [61,62,63]. Consequently, a forward viewing echoendoscope is an alternative to the conventional oblique-viewing echoendoscope

## 5. New Diagnostic Modalities of EUS without Biopsy 

### 5.1. Contrast-Enhanced EUS

In recent years, the utilization of contrast-enhanced EUS (CH-EUS) in the differential diagnosis of SELs has been gathering attention. With the use of contrast agents that contain microbubbles (SonoVue Bracco SpA., Milan, Italy or Sonazoid Daiichi-Sankyo, Tokyo, Japan), CH-EUS is able to provide a detailed view in the microvascularization and the perfusion of the lesions. Then, the examined lesion can be characterized based on the enhancement level. Some studies revealed that CE-EUS is useful for distinguishing between GISTs and benign SELs. Hyperenhancement is suggestive of GISTs with 78–100% sensitivity, 60–100% specificity, and 60–100% accuracy, whereas hypoenhancement is associated with leiomyomas [64,65,66,67]. In addition, a meta-analysis showed that CE-EUS discriminated GISTs from benign SELs with pooled sensitivity and specificity 89% (95% CI 0.82–0.93) and 82% (95% CI 0.66–0.92), respectively [68]. CE-EUS can also be utilized as a method for the estimation of the malignant potential of GISTs. Findings on CE-EUS that are related to high malignant risk include irregular vessels, heterogeneous perfusion pattern, and the presence of nonenhancing spots [69,70,71]. In a meta-analysis of five studies, the pooled sensitivity and specificity of CE-EUS in predicting the malignant risk of GISTs were 96% (95% CI 90–99%) and 53% (95% CI 40–66%), respectively [68]. Consequently, CE-EUS provides useful insights for the nature of the lesion and could be used as an additional, less invasive diagnostic tool to EUS. Thus, lesions that need further evaluation with histological examination can be determined. If a GIST is diagnosed, CE-EUS is also useful for predicting its malignant potential. 

### 5.2. EUS-Elastography

Elastography assesses the stiffness of a certain lesion, which is then reflected as a color spectrum; blue color represents hard lesions, while red color represents soft ones [72]. Elastography is a common procedure for the diagnosis of hepatic, thyroid, and lymph nodes diseases and was recently used as a supplementary tool to EUS for the differentiation of pancreatic lesions and SELs [72]. EUS elastography (EUS-E) can be performed in real time using a conventional EUS probe attached to a processor with specific software installed [73].

In the first pilot study on the effectiveness of EUS-E in the differential diagnosis of gastric SELs, GISTs were harder than other SELs with regard to quality by measuring the amount of stiffness regarding the majority and the distribution of the color [74]. In another study using a strain ratio as an objective marker οf stiffness, EUS-E distinguished GIST from leiomyoma with sensitivity and specificity of 100% and 94.1%, respectively. However, the distinction of GIST from schwannoma was difficult [75]. On the contrary, Guo et al. reported that the utilization of EUS-E in the discrimination of GISTs from leiomyomas could not be supported based on current evidence [76]. 

### 5.3. Artificial Intelligence in Endoscopic Ultrasound 

In recent years, the use of artificial intelligence (AI) has increased in endoscopy. Starting with colonic polyp detection, AI systems now are being tested to determine if they can increase diagnostic accuracy of several endoscopic procedures [77]. So far, 22 articles have been published on the use of AI in endosonography; six deal with gastric SELs. Minoda et al. demonstrated that AI has a better accuracy, sensitivity, and specificity in recognizing GIST tumors from non-GIST lesions independent of the lesion size in comparison to senior endoscopists. In comparison to histology, the AI system had an accuracy, sensitivity, and specificity of 86.3, 86.3, and 62.5%, respectively, for lesions < 2 cm, and 90.0, 91.7, and 83.3% for lesions > 2 cm [78]. These results are comparable with the results of FNB. Therefore, the authors of this study concluded that EUS-AI can be an alternative to tissue sampling when differentiating GIST lesions from non-GIST. Hirai et al. and Kim et al. found similar results [79,80]. Seven at al. demonstrated that AI has a high accuracy in predicting the malignant potential of GIST tumors (low vs. high risk) up to 99% [81]. Additionally, Kim et al. were able to differentiate in non-GIST lesions leiomyomas from schwannomas in 75% [80]. Consequently, AI systems seem to be a promising additional tool when differentiating gastric SELs, but more studies are required for its validation. 

### 5.4. Critical Appraisal of the Evidence

So far, these new diagnostic advances in EUS (CE-EUS, EUS-E, and EUS-AI) are at a research level and are not broadly clinically available. Currently, the European guidelines suggest to use CE-EUS for lesion characterization and determination of its malignant potential, if available. Nevertheless, tissue acquisition is still necessary [4]. As far as EUS-E is concerned, contradictory results have been published so far. Therefore, at the moment, there are not enough data yet to recommend or to reject this method for the diagnosis of gastric SELs [4]. Similarly, more studies are needed for the use of AI in EUS-guided diagnosis of gastric SELs. Interestingly, the American guidelines did not make any reference to the first two methods in their latest version, apart from a short reference to the use of AI [2]. 

For all these new diagnostic modalities we need more data. Firstly, we need large-scale studies where a greater amount of diverse gastric subepithelial lesions will be characterized and assessed by these new technologies. Furthermore, we need prospective multicentric studies that will evaluate the sensitivity, specificity, and accuracy of these techniques alone as well as in combination with each other. Additionally, the interobserver variability needs to examined, along with the applicability and diagnostic accuracy of these methods when used by nonexperts in the respective method. 

## 6. The Role of Cross-Sectional Imaging in the Diagnosis of Gastric SELs 

Last but not least, we wanted to assess if diagnostic procedures other than EUS play a role in the differentiation of gastric SELs. Computed tomography (CT) is a very valuable imaging technique in the evaluation of abdominal diseases. With regard to SELs, the ability of CT to discriminate GISTs from other SELs is dependent on the tumor size, with lesions < 10 mm having the highest probability of being undetectable [82,83,84]. Additionally, two retrospective studies compared the diagnostic yield of CT with EUS, having histopathological diagnosis as reference. In the first study, in 71 SELs smaller than 5 cm, CT showed comparable accuracy with EUS (78.9% vs. 74.6%) [85]. However, Kim et al. found that EUS outperformed CT in the diagnosis of GISTs, leiomyomas, and ectopic pancreas with overall accuracy of 64.2% vs. 50.9%, respectively [86]. This inconsistent performance, coupled with the possibility of EUS to be accompanied by tissue sampling for analysis, make EUS the best approach for the diagnostic evaluation of SELs. However, in the case of bigger SELs, CT scan can be used for disease staging. 

The role of PET/CT in the differential diagnosis of gastric subepithelial lesions is limited. Although it can differentiate, with 80% sensitivity and 66.7% specificity, low-risk GISTs from high-risk GISTs [87], GISTs cannot be distinguished from leiomyomas and schwannomas, since they also demonstrate an increased signal [87]. However, PET/CT can be used for staging of GISTs and neuroendocrine tumors (with the exception of Type 1 NETs of the stomach) [16] and can also be used for treatment response evaluation [88]. Therefore, current guidelines do not recommend PET/CT for differential diagnosis but for treatment planning [16].

## 7. Follow-Up 

In cases where definite diagnosis has been made, the necessity of surveillance is divergent for each lesion. For benign lesions such as leiomyoma, lipoma, schwannoma, pancreatic rest, and others, surveillance is not required.

Regarding GISTs, the management after detection is more complicated. The malignant potential of GISTs is determined by their size, mitotic activity, and the location of the tumor (GISTs occurring in the stomach are associated with a lower malignant potential) [89]. Since the mitotic activity is best defined after resection, tumor size is a critical factor for their further management. For GISTs > 2 cm in size or with high-risk EUS features (irregular border, echogenic foci, cystic spaces), several guidelines recommend resection [2,4]. Conversely, for GISTs < 2 cm, surveillance seems a reliable approach as the risk of progression and metastasis for these tumors is extremely low [2,4,90]. Nevertheless, European Society for Medical Oncology (ESMO) suggests resection regardless of size [78]. If surveillance is chosen, there are different recommendations about the follow-up strategy which the clinician can follow. Most experts agree with an initial strict follow-up interval (e.g., EUS at 3–6 months) in order to assess the growth of the lesion [4,91,92]. Then, surveillance with EUS at intervals of 1–2 years for lesions with size of 1–2 cm and at intervals of 2–3 years for lesions less than 1 cm is reasonable [4,92]. 

In cases without a definite diagnosis, for example, hypoechoic, asymptomatic SELs < 2 cm without high-risk features on EUS, tissue sampling for histological examination is not mandatory [2]. In fact, these types of tumors carry a very low risk of size increment (<4% in the range of 16–118 months), making surveillance an appropriate option for them [4,93,94]. However, one needs to keep in mind that these tumors still can be GISTS with inherent malignant potential, so the compliance with surveillance is of great importance. 

Surveillance should be performed using EUS, because only EUS can assess high-risk features [4]. However, at least two research groups have shown that TAUS could be an option for follow-up as a noninvasive and cost-effective method. Liu et al. studied the diagnostic accuracy of TAUS after administration of an oral-echoic-cellulose-based gastric contrast agent [95], while Tsai et al. assessed the same after oral administration of water only [96]. Lesions smaller than 10 mm, especially in cardia and fundus and in obese patients, were more likely to be missed, whereas lesions in the body and antrum and lesions > 1 cm were identified at 100% and 94%, respectively. Experienced sonographers could localize the layer of origin, but further differentiation of the lesions was not possible through TAUS [95,96]. Currently, both the European and the American guidelines do not mention transabdominal ultrasound (TAUS) as an option for surveillance. 

## 8. Future Directions

An array of new diagnostic modalities (CE-EUS, EUS-E, EUS-AI) has been recently developed for the assessment of subepithelial lesions. In the following years, these diagnostic exams will be further improved and evaluated in multicentric studies with bigger cohorts. Thus, all lesions, even the less common ones, will be characterized and standardized. Consequently, we anticipate that these new methods will become an additional (at least optional) assisting step along the diagnostic algorithm for the differentiation of gastric SELs or even circumvent EUS-guided biopsy in certain cases. At the same time, we experience an increase in the number of resection procedures such as endoscopic submucosal dissection and full thickness resection. With these techniques, a direct treatment is feasible; thus, they could reduce the need for accurate pre-resection diagnoses at least in certain cases and will affect current surveillance strategies, especially in cases without a definite diagnosis. 

## Figures and Tables

**Table 1:** Overview of the most common gastric SELs. NET: neuroendocrine tumor (adjusted from [2,4]).

SEL Type	Origin	EUS Morphology	Border	Location in Stomach	Malignant Potential	Biopsy Needed
Duplication cyst	3rd/ external	Anechoic, no Doppler signal	Sharp	Any	Very Rarely	No
Varices	3rd	Anechoic with Doppler signal	Sharp, serpiginous shape	Any	No	No
Lymphangiomas	3rd	Anechoic, no Doppler signal, with internal septa	Sharp	Any	No	Yes
Lipoma	3rd	Hyperechoic, homogenous	Sharp	Any	No	No
Glomus Tumor	3rd/4th	Hypo/Hyperechoic Hypervascular with internal echo	Sharp	Any	Rarely	Yes
NET	1st/2nd 3rd	Hypoechoic/hyperechoic	Sharp	Any	Yes	Yes
GIST low risk	2nd/4th	Hypoechoic, heterogenous, hypervascular	Sharp when benign	Any	Yes	Yes
GIST high risk	2nd/4th	Hypoechoic, heterogeneous with cystic space or echogenic foci	Irregular > 3 cm	Any	Yes	Yes
Leiomyoma	2nd/4th	Hypoechoic, rarely multifocal	Sharp	Cardia	No	Yes
Lymphoma	2nd/3rd/4th	Hypoechoic	Irregular	Any	Malignant	Yes
Inflammatory fibroid Polyp	2nd/3rd	Hypoechoic, homogenous, polypoid	Indistinct	Antrum	No	Yes
Ectopic pancreas	3rd/4th	Hypoechoic, heterogenous, with cysts or ducts inside	Indistinct	Antrum	Very rarely	Sometimes
Schwannoma	4th	Hypoechoic, homogenous, sometimes with marginal halo	Sharp	Body	No	Yes
Metastasis	Any	Hypoechoic	Irregular	Any	Malignant	Yes

## Data Availability

Not applicable.
